# Transcriptional regulation of normal human mammary cell heterogeneity and its perturbation in breast cancer

**DOI:** 10.15252/embj.2018100330

**Published:** 2019-01-11

**Authors:** Davide Pellacani, Susanna Tan, Sylvain Lefort, Connie J Eaves

**Affiliations:** ^1^ Terry Fox Laboratory British Columbia Cancer Agency Vancouver BC Canada

**Keywords:** breast cancer, chromatin, epigenomics, mammary, transcriptional regulation, Cancer, Transcription

## Abstract

The mammary gland in adult women consists of biologically distinct cell types that differ in their surface phenotypes. Isolation and molecular characterization of these subpopulations of mammary cells have provided extensive insights into their different transcriptional programs and regulation. This information is now serving as a baseline for interpreting the heterogeneous features of human breast cancers. Examination of breast cancer mutational profiles further indicates that most have undergone a complex evolutionary process even before being detected. The consequent intra‐tumoral as well as inter‐tumoral heterogeneity of these cancers thus poses major challenges to deriving information from early and hence likely pervasive changes in potential therapeutic interest. Recently described reproducible and efficient methods for generating human breast cancers *de novo* in immunodeficient mice transplanted with genetically altered *primary* cells now offer a promising alternative to investigate initial stages of human breast cancer development. In this review, we summarize current knowledge about key transcriptional regulatory processes operative in these partially characterized subpopulations of normal human mammary cells and effects of disrupting these processes in experimentally produced human breast cancers.

## The normal adult human mammary gland

**Figure 1:**
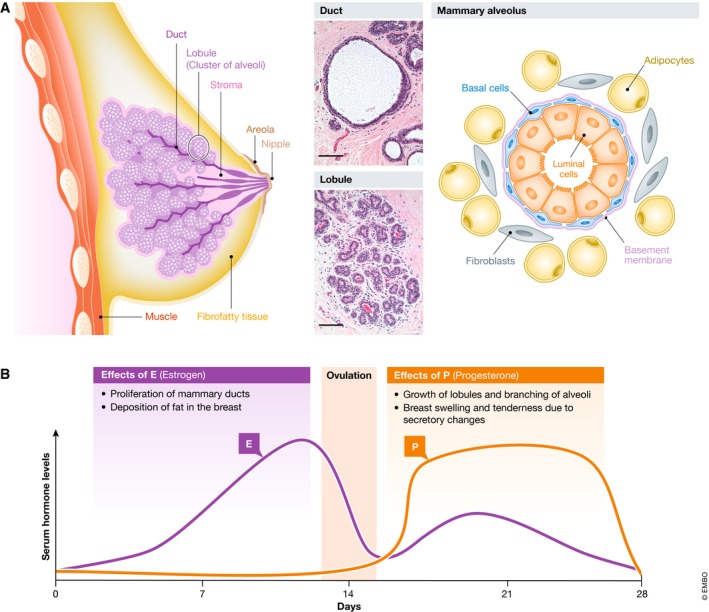
Macro‐ and microscopic structure of the normal human breast (A) Diagram showing the macroscopic structure of the human breast and histological sections of ducts and alveoli (scale bar = 100 μm). (B) Effects of serum hormone levels on the human mammary epithelium during the menstrual cycle.

The adult human female mammary gland is a continuous branching tree of ducts that extend radially from the nipple and terminate in expanded alveolar structures frequently called lobules (Fig 1A). This structure is encased in a basement membrane and an outer layer of fibroblasts, all of which are embedded in a collagen‐rich stroma containing adipocytes, macrophages, lymphocytes, and blood and lymph vessels. The mammary gland, itself, consists of two layers of cells with different features and functions. The outer “basal” layer is made up of cells that are in direct contact with the basement membrane. These cells are also referred to as myoepithelial cells because they possess contractile, smooth muscle‐like properties. The inner “luminal” layer of the gland contains cells with quite different, polarized epithelial features and an ability to produce and secrete milk upon hormone induction.

The initial stages of development of the mammary gland that take place in humans before birth are not well documented, and hence, knowledge of these has had to rely on inferences drawn from studies of mice (Veltmaat *et* *al,* 2003; Spike *et* *al,* 2012; Makarem *et* *al,* 2013b). In that species, the mammary gland can be seen to originate in the embryo from cells in the ventral ectoderm that invade the underlying mesoderm to form a primitive branching structure. At this stage, the rudimentary gland is composed of cells with a mixture of properties that are associated with distinct cell types found in the adult mouse mammary gland. This primitive structure then expands rapidly after the onset of puberty. Thereafter, until menopause, the entire mammary gland in humans and mice alike undergoes continuous cyclical phases of expansion and involution under the control of changing levels of estrogen (E) and progesterone (P) (Fig 1B; Ramakrishnan *et* *al,* 2002). Current evidence indicates that the stimulatory effects of these hormones are exerted indirectly by activating paracrine signaling mechanisms that involve an upregulated production of amphiregulin by E, an induced secretion of RANKL by P, and an enhancing effect of hormonally controlled changes by WNT‐producing macrophages (Wilson *et* *al,* 2006; Asselin‐Labat *et* *al,* 2010; Brisken & O'Malley, 2010; Joshi *et* *al,* 2010; Roarty & Rosen, 2010; Visvader & Stingl, 2014; Arendt & Kuperwasser, 2015; Chakrabarti *et* *al,* 2018). Other growth factors implicated in regulating mammary gland development and homeostasis include members of the epidermal growth factor (EGF), insulin‐like growth factor (IGF), and fibroblast growth factor (FGF) families (Hynes & Watson, 2010).

**Figure 2:**
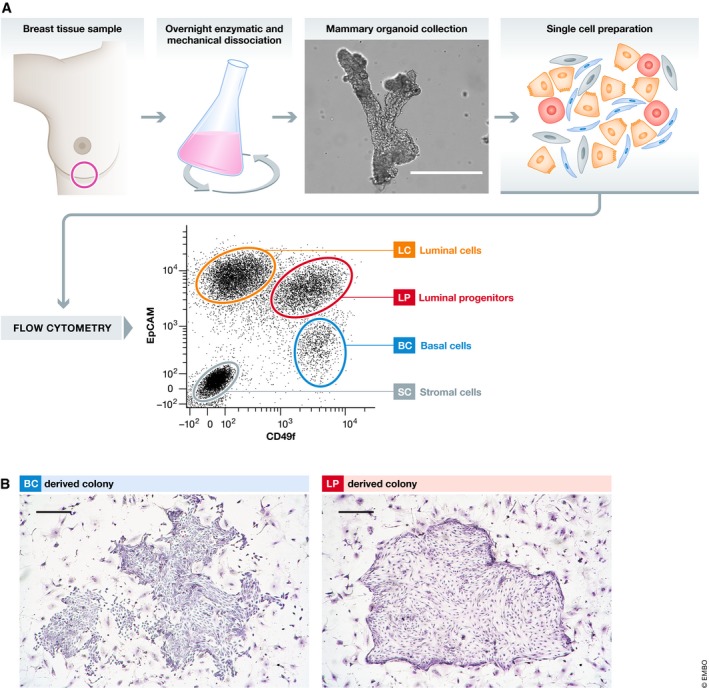
Subpopulations of cells within the normal adult human mammary gland (A) Diagram showing the workflow for separating the four main cell populations present in the breast in addition to blood cells and endothelial cells (scale bar = 400 μm). (B) Examples of typical Giemsa‐stained colonies derived from BCs and LPs and assessed after 7–9 days *in vitro* (scale bar = 400 μm).

The development of reproducible methods for isolating the different cell types that constitute the major components of the normal adult human mammary gland as separate suspensions of single viable cells was a key advance because it then enabled the further biological and molecular characterization of these different cell types. Most studies of normal human mammary cells have made use of discarded tissue obtained from women without known breast disease undergoing reduction mammoplasties. The pieces of tissue obtained are then subjected to a series of enzymatic dissociation and filtration steps, followed by removal of prevalent blood and endothelial cells using antibodies against CD45 and CD31. The three major cell types that constitute the mammary gland, plus remaining stromal fibroblasts, can then be separately isolated using flow cytometry according to their differential staining with antibodies to CD49f and EpCAM (Fig 2A). The three subpopulations of mammary cells obtained are typically referred to as basal cells (BCs), luminal progenitors (LPs), and luminal cells (LCs). Other antibody cocktails have also been used to obtain highly overlapping phenotypes with very similar biological and molecular properties (Raouf *et* *al,* 2008; Bachelard‐Cascales *et* *al,* 2010; Keller *et* *al,* 2012; Kannan *et* *al,* 2014; Nguyen *et* *al,* 2014; Fridriksdottir *et* *al,* 2015; Lawson *et* *al,* 2015; Britschgi *et* *al,* 2017), and additional markers have proven useful to subdivide these three subpopulations of human mammary cells even further (Eirew *et* *al,* 2012; Shehata *et* *al,* 2012; Knapp *et* *al,* 2017; Morel *et* *al,* 2017). However, the combination of antibodies to CD49f and EpCAM has generally been the most widely utilized.

BCs are defined by their CD49f^+^EpCAM^low^ phenotype and are so‐named because they express numerous markers (e.g., KRT14, TP63, ACTA2/SMA, MME/CD10, and THY1/CD90) that distinguish cells of the basal layer from those of the luminal layer in histological preparations of normal human mammary tissue. In culture media containing insulin and EGF, as well as other supplements and a feeder layer of fibroblasts, ~ 10–20% of freshly isolated BCs plated at low density will produce readily visualized adherent colonies within 8–10 days (Fig 2B; Eirew *et* *al,* 2008; Kannan *et* *al,* 2013). Many of the individual colonies produced from BCs under these conditions will contain a mixture of cells expressing either basal or luminal markers (Stingl *et* *al,* 2001). A smaller fraction of the BCs (~ 0.1%) will produce bilayered epithelial structures that resemble the normal human mammary gland when injected directly into “humanized” fat pads (Kuperwasser *et* *al,* 2004; Proia & Kuperwasser, 2006; Lim *et* *al,* 2009) or when transplanted in collagen gels that are then inserted either under the kidney capsule (Eirew *et* *al,* 2008, 2010; Nguyen *et* *al,* 2015) or subcutaneously (Pellacani D and Eaves C, unpublished) in immunocompromised mice. In both of these sites, the regenerated human mammary gland‐like structures contain the same spectrum of EGF‐dependent *in vitro* mammary colony‐forming cells (CFCs) that are present in the normal human mammary gland, as well as rarer cells that can regenerate similar bilayered mammary gland structures and mammary CFCs upon transplantation into secondary hosts (Eirew *et* *al,* 2008; Lim *et* *al,* 2009; Nguyen *et* *al,* 2014). In addition, the regenerated human gland‐like structures will produce human milk proteins when appropriately hormonally stimulated (Eirew *et* *al,* 2008).

LPs and LCs are defined by their shared high expression of EpCAM, a well‐established marker of cells that constitute the luminal layer of mammary glands. Both LPs and LCs also express other markers histologically associated with the luminal layer (e.g., KRT8, KRT18, and MUC1). However, these EpCAM^+^ mammary cells can be readily subdivided according to their differential expression of CD49f (and CD117, c‐KIT). LC is the term assigned to the CD49f^−^ cells within the EpCAM^+^ fraction, and they include most of the cells that express E and P receptors (ER/ESR1 and PR/PGR) and express low to undetectable levels of EGFR (Lim *et* *al,* 2009). Not surprisingly, LCs do not mount a significant direct signaling response to EGF (Knapp *et* *al,* 2017) and do not proliferate when exposed to EGF *in vitro* (Kannan *et* *al,* 2013, 2014). They are also incapable of reconstituting epithelial structures *in vivo* that contain clonogenic progeny (Eirew *et* *al,* 2008). However, it was recently reported that a small proportion (~ 0.4%) of EpCAM^+^CD271^−^CD166^high^CD117^low^ human mammary cells, a phenotype expected to overlap with CD49f^−^EpCAM^hi^ LCs, will form colonies in cultures containing inhibitors to the TGF‐β pathway (Fridriksdottir *et* *al,* 2015). Interestingly, cultures established from these cells could be expanded for 15 population doublings and their progeny continued to express ER and respond to E stimulation. In mice, similar evidence of the proliferative activity *in vivo* of non‐clonogenic LCs has also been obtained from BrdU incorporation studies (Giraddi *et* *al,* 2015). Together, these findings raise the possibility that at least some human mammary cells with a LC phenotype can proliferate when appropriately stimulated. Nevertheless, the relevance of these *in vitro* findings to events that underpin the cellular dynamics within the mammary gland of normal adult women remains obscure as, *in situ*, very few ER^+^ or PR^+^ mammary cells appear to be proliferating (Clarke *et* *al,* 1997; Stingl, 2011).

LPs are defined as the EpCAM^+^ cells that co‐express CD49f, suggesting that they might be an intermediate stage between BCs and LCs. However, these cells express other markers specific to the luminal layer of the epithelium assessed histologically, although only a minority express ER or PR (Lim *et* *al,* 2009). LPs are also distinct in their expression of high levels of CD117, a marker often used for their differential isolation (Fridriksdottir *et* *al,* 2015; Lawson *et* *al,* 2015). Approximately 50% of LPs also express KRT5/6 (Lim *et* *al,* 2009), a type of cytokeratin known to be expressed by cells in the basal layer of many types of epithelia (Purkis *et* *al,* 1990; Böcker *et* *al,* 1992). On average, 20–30% of LPs will generate colonies *in vitro* under the same conditions as BCs (Fig 2B). But, in this case, only cells with luminal features are produced (Stingl *et* *al,* 2005). A small proportion of LPs have also been reported to regenerate epithelial structures *in vivo* (Shehata *et* *al,* 2012), but the structures produced do not contain CFCs (Eirew *et* *al,* 2008).

Most LPs have very short telomeres and display a pronounced telomere‐associated DNA damage response, even in mammary cells obtained from women in their twenties (Kannan *et* *al,* 2013). Interestingly, some LPs expressing activated caspase‐3 will still show considerable subsequent proliferative activity *in vitro* (Knapp *et* *al,* 2017). LPs are also distinguished by elevated levels of reactive oxygen species (ROS) compared to LCs and BCs. In addition, they display an innately greater resistance to oxidative stress and a higher level of associated DNA damage (Kannan *et* *al,* 2014), two processes that have been proposed to accelerate telomere shortening (von Zglinicki, 2002; Richter & von Zglinicki, 2007), and predispose cells to transformation.

More recently, single‐cell mass cytometry (Knapp *et* *al,* 2017) and RNA sequencing methodologies (Nguyen *et* *al,* 2018) have provided further support for the segregation of normal human mammary epithelial cells into the same three main cell types. On the other hand, these studies have also highlighted their extensive molecular heterogeneity and the possible existence of new subsets within each (Shehata *et* *al,* 2012; Knapp *et* *al,* 2017; Nguyen *et* *al,* 2018). Nevertheless, pseudo‐temporal ordering of the available single‐cell transcriptional data produces a differentiation trajectory profile that separates into three main branches corresponding to the historically visualized distinction of cells produced in the normal adult human mammary gland (Nguyen *et* *al,* 2018).

Taken together, these findings are consistent with a hierarchically organized sequence of changes initiated in bipotent BCs that are able to generate progeny with either luminal or basal features. Cells with luminal features can then be phenotypically and biologically segregated into an intermediate, luminal‐restricted but EGF‐responsive state, and a state in which the capacity to proliferate in response to EGF has been lost. However, this model of a hierarchical differentiation process should not be viewed as necessarily reflecting a series of tightly co‐ordinated events and may also not reflect the operation of mechanisms that maintain these subpopulations under normal homeostatic conditions. Indeed, in the mouse, where analogous populations of BCs, LPs, and LCs have been identified, some luminal cells possess or can acquire the regenerative activity originally thought to be restricted to BCs (Shehata *et* *al,* 2012; Makarem *et* *al,* 2013a). In addition, in mice, *in situ* lineage‐tracing experiments suggest that both myoepithelial and luminal lineages can display self‐sustaining dynamics (Van Keymeulen *et* *al,* 2011), despite the continued presence and activity of transplantable cells with the bipotent regenerative properties of “stem cells” (Rios *et* *al,* 2014). Such findings are consistent with increasing evidence of an incomplete overlap of mechanisms that control mammary cell proliferative potential and those that determine whether their differentiated state will change (or not) with sequential divisions.

At the same time, it is important to recognize the caveats and assumptions inherent in available methods for associating functional and molecular properties of individual human mammary cells or the history of their acquisition and display. Deriving these associations is necessarily limited by an inability to undertake the requisite prospective lineage‐tracing experiments in humans. Accordingly, direct measurements of normal human mammary cell outputs *in situ* cannot be compared with the outputs that can be elicited from the same cells when they are exposed to highly stimulatory conditions *in vitro* or following their transplantation into mice. In addition, both flow cytometry and clonal assays have technical limitations of efficiency and specificity. They may also be compromised by the use of markers that are not co‐ordinately controlled by mechanisms that regulate their functional properties. However, these caveats may be partially reduced by the use of index‐sorting strategies to link molecular and functional properties more directly (Wilson *et* *al,* 2015), thereby circumventing the problem of assigning functions of rare cells present in bulk isolates.

## Transcriptional differences between human mammary cell subsets

A variety of technologies have been used over the past 10 years to characterize the transcriptomes of BCs, LPs, and LCs isolated from normal adult female breast tissue (Bloushtain‐Qimron *et* *al,* 2008; Raouf *et* *al,* 2008; Lim *et* *al,* 2009, 2010; Maruyama *et* *al,* 2011; Shehata *et* *al,* 2012; Kannan *et* *al,* 2013; Gascard *et* *al,* 2015; Pellacani *et* *al,* 2016). These studies have revealed consistent differences in the activity of hundreds of genes in each of these phenotypically defined subsets. In turn, these studies have pointed to a number of differentially activated pathways that may regulate their different biological properties (Liu *et* *al,* 2005). For example, many components of the NOTCH pathway are expressed at different levels in BCs, LPs, and LCs, with some evidence of corresponding functional consequences (Dontu *et* *al,* 2004; Raouf *et* *al,* 2008). WNT pathway components also show differential patterns of expression, with biological evidence of their importance in maintaining a mammary stem cell state, at least as inferred from studies of the mouse mammary gland (Teulière *et* *al,* 2005; Roarty & Rosen, 2010; Zeng & Nusse, 2010; van Amerongen *et* *al,* 2012; Gu *et* *al,* 2013) with more limited, but consistent data for human cells (Arendt *et* *al,* 2014). Other pathways similarly implicated are the TGF‐β (Moses & Barcellos‐Hoff, 2011; Kahata *et* *al,* 2017) and the Hippo pathways (Chen *et* *al,* 2014; Pelissier *et* *al,* 2014; Skibinski *et* *al,* 2014; Shi *et* *al,* 2015; Britschgi *et* *al,* 2017). Importantly, all of these are variably deregulated in breast cancers (Howard & Ashworth, 2006).

## Human mammary cell epigenomes reflect their transcriptional profiles

**Figure 3:**
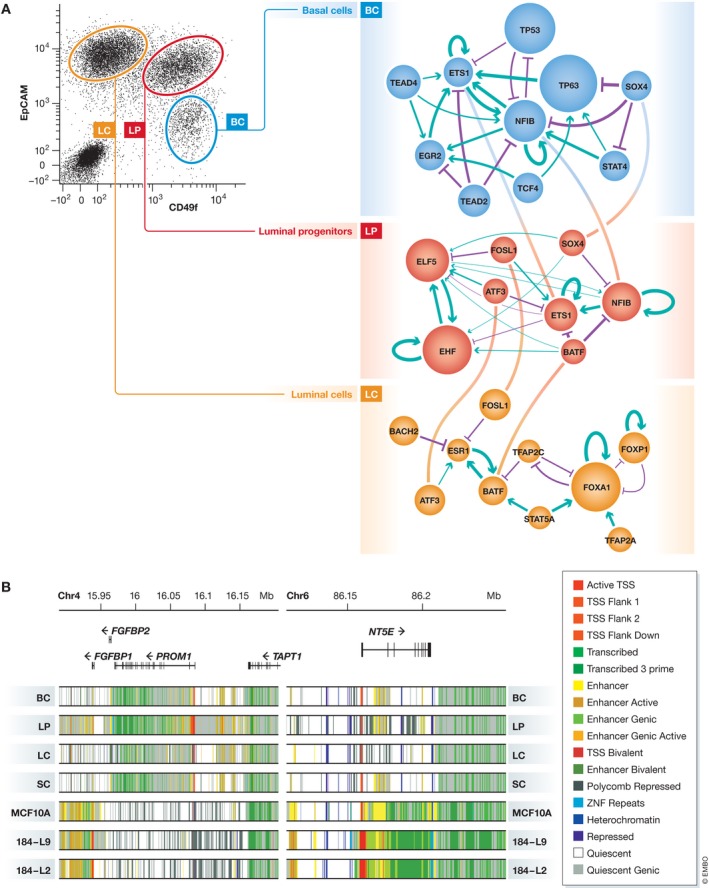
Transcriptional regulation of normal human mammary cell subpopulations (A) TF regulatory networks constructed from the chromatin profiles at enhancers of BCs, LPs, and LCs. (B) Genome browser plots showing the differences in chromatin states defined for normal human mammary cell subpopulations and non‐tumorigenic mammary cell lines around the *PROM1* and the *NT5E* genes.

Several studies have now characterized the epigenomic features of human as well as mouse mammary cells (Maruyama *et* *al,* 2011; Choudhury *et* *al,* 2013; Dos Santos *et al,* 2015; Gascard *et* *al,* 2015; Huh *et* *al,* 2015; Pellacani *et* *al,* 2016; Shin *et* *al,* 2016; Lee *et* *al,* 2017). Early studies reported an association of differences in the H3K27me3 and DNA methylation of genes that are differently expressed in luminal and basal subsets (Maruyama *et* *al,* 2011). These genes include several that encode transcriptional regulators and/or other members of pathways of reported activity in the mammary gland. Subsequent analyses revealed DNA methylation to be a stable mark of exonic and intronic usage, with evidence of intron retention events specific to each subpopulation and linked to differences in protein expression (Gascard *et* *al,* 2015). The latter study also found many more hypo‐methylated enhancer elements in luminal cells (LPs + LCs) than in BCs and these were commonly associated with binding sites for *FOXA1*,* GATA3,* and *ZNF217*. These studies also indicated a higher overall transcriptional activity in the luminal cells. More extensive epigenomic characterization of highly purified human BCs, LPs, LCs and their associated stromal cells has now been derived from ChIP‐seq analyses of H3K4me1, H3K4me3, H3K27me3, H3K27ac, H3K36me3, and H3K9me3 marks on histones and accompanying whole‐genome bisulfite sequencing, with matching mRNA‐seq and miRNA‐seq data for the same cells (Pellacani *et* *al,* 2016). From these datasets, the chromatin landscape at putative enhancer sites of these different mammary cell types has been derived. Comparisons of these have also shown LPs to be intermediate between BCs and LCs, consistent with their different biological properties. Analysis of transcription factor binding sites (TFBS) and derived TF networks for each subpopulation has also enabled novel TFs to be identified as potential regulators of each subpopulation, in addition to others previously reported. Analysis of our more recently accrued epigenomic data has also provided new evidence of a bipartite TF network in LPs that includes elements of those operative in BCs and LCs (Fig 3A). In addition, this study showed that the epigenomic and transcriptional profiles of primary sources of normal human mammary cells are very different from those of established lines of immortalized but non‐tumorigenic mammary cells (Fig 3B; Pellacani *et* *al,* 2016). This latter finding highlights the caveats of relying on data from such immortalized cell lines to infer mechanisms controlling the biological properties of normal human mammary cells, and, conversely, the importance of analyzing primary isolates for this purpose.

## Epigenomic and transcriptional changes related to aging and reproductive history

Aging and pregnancy are associated, respectively, with an increase and decrease in breast cancer risk. Several groups have therefore started dissecting the molecular changes evident in mammary cells obtained from donors of different ages or different reproductive histories. These include a report of an expansion with aging of defective multipotent progenitors that show altered interactions with extracellular matrix elements and in KRT14^+^ and CD49f^+^ luminal cells (Garbe *et* *al,* 2012; Pelissier *et* *al,* 2014). Accompanying transcriptome changes suggested an aging‐associated epigenomic deregulation, potentially mediated by changes in the microenvironment of the mammary gland (Miyano *et* *al,* 2017). Comparison of the transcriptomes of purified mammary cell subsets isolated from breast tissue of parous and nulliparous women has shown differences between the CD44^+^ cells from these two sources, with *CDKN1B* (p27) as one of the most differentially expressed genes (Choudhury *et* *al,* 2013). More extensive studies in mice have shown pregnancy to be associated with long‐lasting alterations in DNA methylation profiles at sequences enriched for STAT5 binding sites (Dos Santos *et al,* 2015).

## Transcription factors regulating normal mammary cells

**Figure 4:**
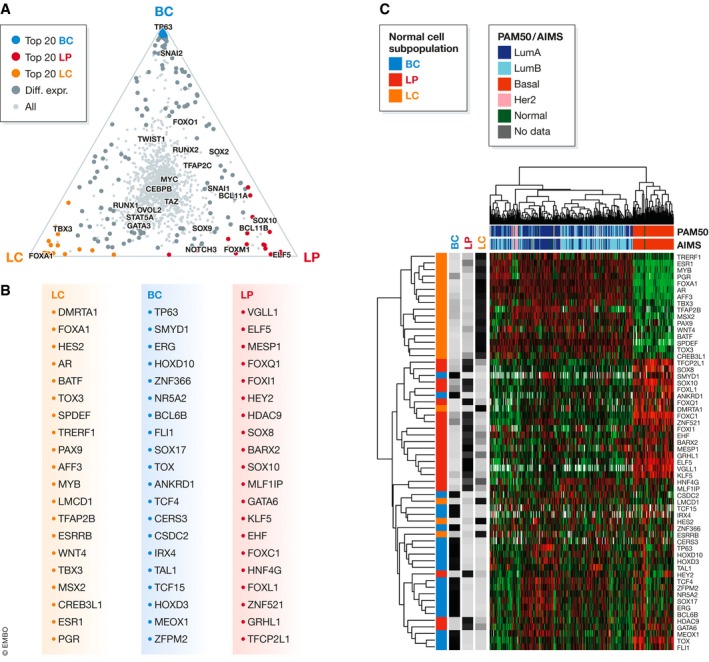
Transcriptional regulators active in the normal human mammary gland and in human breast cancer (A) Ternary plot of relative expression of all transcriptional regulators in normal human mammary cell subpopulations from a re‐analysis of the RNA‐seq data presented in Pellacani *et* *al* (2016). Transcriptional regulators discussed in the text are highlighted. (B) List of the top 20 transcriptional regulators most specific to each cell type highlighted in (A). (C) Clustering of the tumors profiled by RNA‐seq in Nik‐Zainal *et* *al* (2016) using the genes shown in (B).

Epigenomic and transcriptional profiling of primary human mammary cells has also led to the identification of many candidate TFs that show subpopulation specificity. For example, several TFs are significantly elevated in only one of the three major subpopulations of normal human mammary cells (Fig 4A–C). *In silico* predictions further identify a differential enrichment of associated TFBSs at epigenetically defined promoter and enhancer regions in these cell types (Lim *et* *al,* 2010; Kannan *et* *al,* 2013; Gascard *et* *al,* 2015; Pellacani *et* *al,* 2016). Several studies in mice or human cell lines have also implicated a multitude of TFs to be involved in mammary cell development and differentiation. However, similar analyses of primary human cells are still very limited, although the strong correlations found between *in silico* predictions and results obtained from mice justify a brief overview of these.

One of the TFs implicated in modulating mouse mammary stem cell activity by acting directly on BCs is ∆Np63, a known regulator of normal stem cell maintenance in multiple epithelial tissues (Senoo *et* *al,* 2007). ∆Np63 appears to act by modulating several key pathways. These include enhancing WNT signaling by upregulating *Fzd7* expression (Chakrabarti *et* *al,* 2014), activating Hedgehog signaling (Li *et* *al,* 2008; Memmi *et* *al,* 2015), and partially counteracting the effects of Notch signaling (Yalcin‐Ozuysal *et* *al,* 2010). TP63 expression in basal cells is also necessary during pregnancy and lactation: Genetic deletion of *Trp63* in keratin 14‐expressing cells of the adult mouse leads to defects in luminal cell proliferation and differentiation, and failure to produce milk, due to lack of expression of the EGF family ligand NRG1 in basal cells which is required for ERBB4/STAT5A activation in luminal cells (Forster *et* *al,* 2014). Several SOX family TFs have likewise been implicated. For example, modulation of SOX9 expression was found to directly influence the ability of mouse mammary cells to produce organoid structures *in vitro* (Guo *et* *al,* 2012) and its conditional knockout impaired postnatal development of the gland (Malhotra *et* *al,* 2014). SOX10 is expressed specifically in mammary cells exhibiting the highest levels of stem/progenitor activity (Dravis *et* *al,* 2015) and SOX2 has also been implicated, albeit less directly, as its expression was induced by LGR4 downstream of WNT signaling (Wang *et* *al,* 2013).

Many of these studies in mice have associated expression of SOX TFs with the acquisition of features characteristic of mesenchymal cells in a process resembling an embryonic epithelial–mesenchymal transition (EMT). In fact, the possession of mesenchymal features has been frequently associated with mammary stem cell activity, both during development and subsequently throughout adulthood (Mani *et* *al,* 2008; Guen *et* *al,* 2017), although this is still controversial (Sikandar *et* *al,* 2017). Nevertheless, many other TFs associated with EMT have been directly linked to changes in the clonogenic or repopulating activity of mouse mammary cells. Of these, SNAI2 (SLUG) has been reported to cooperate with SOX9 (Guo *et* *al,* 2012) in regulating the transition of mouse mammary stem cells to short‐term progenitors (Phillips *et* *al,* 2014). SNAI1 (SNAIL) is another member of this group, and it was found to regulate the spindle orientation machinery in mammary stem cells responding to SLIT2/ROBO1 signaling (Ballard *et* *al,* 2015). OVOL2, a transcriptional repressor, was likewise reported to restrict activation of EMT (Watanabe *et* *al,* 2014). More recently, another transcription factor, ZEB1, was shown to be expressed at high levels in a fraction of mammary BCs (Nguyen *et* *al,* 2018) and associated with cells expressing protein C receptor (ProCR; Wang *et* *al,* 2015). ZEB1 was also recently reported to have a protective role against oncogene‐induced DNA damage in normal human mammary epithelial cells (Morel *et* *al,* 2017). Other TFs involved in mammary stem cell function include FOXO1 (Sreekumar *et* *al,* 2017), RUNX2 (Ferrari *et* *al,* 2015), MYC (Hynes & Stoelzle, 2009; Moumen *et* *al,* 2012), CEBPB (C/EBPβ; LaMarca *et* *al,* 2010), BCL11A (Khaled *et* *al,* 2015), and BCL11B (Miller *et* *al,* 2018).

TFs implicated in regulating luminal cell production and maintenance have also been identified. Of these, GATA3 was found to have an essential role in controlling the morphogenesis of the mammary gland in the mouse embryo, during puberty, and in adult life (Kouros‐Mehr *et* *al,* 2006; Asselin‐Labat *et* *al,* 2007). In addition, GATA3 promoted differentiation of cells within the luminal lineage in mice, potentially through a positive regulatory loop with ESR1 (Eeckhoute *et* *al,* 2007). FOXA1 was found to be involved in hormone‐induced mammary ductal invasion (Bernardo *et* *al,* 2010), but did not affect lobulo‐alveolar maturation and milk production. ELF5 was shown to be necessary for alveologenesis during pregnancy (Choi *et* *al,* 2009), and its deletion led to an accumulation of cells with mixed basal/luminal molecular phenotypes (Chakrabarti *et* *al,* 2012b). ELF5 was found to suppress EMT by down‐regulating transcription of SNAI2 (Chakrabarti *et* *al,* 2012a). ELF5 also acted directly in LPs (Yamaji *et* *al,* 2009) to influence expression of STAT5A (Choi *et* *al,* 2009), another TF involved in alveologenesis (Liu *et* *al,* 1997). Contrary to the effects of RUNX2, RUNX1 was shown to induce the appearance of ER^+^ luminal cells at least partially through the modulation of *ELF5* and *FOXA1* expression (van Bragt *et* *al,* 2014), potentially downstream of the p38α kinase (Del Barco Barrantes *et* *al,* 2018).

Notably, the Hippo pathway regulator TAZ, together with many other TFs, has recently emerged as a negative regulator of luminal differentiation in primary human cells (Skibinski *et* *al,* 2014). Other TFs and chromatin modifiers necessary for correct human luminal cell differentiation include TFAP2C (Cyr *et* *al,* 2015), TBX3 (Arendt *et* *al,* 2014), NOTCH3 (Raouf *et* *al,* 2008), FOXM1 (Carr *et* *al,* 2012), and KDM6A (Yoo *et* *al,* 2016). However, many “potential” TFs identified more recently from genome‐wide epigenomic analyses of both human and mouse mammary gland cells remain poorly characterized.

## Cellular and molecular heterogeneity of human breast cancers

Breast cancers arise from single cells as aberrant clones of progeny that undergo a continuous process of evolution, demarcated by distributed genetic and epigenetic alterations in successive generations of daughter cells (Balani *et* *al,* 2017). Those that maintain and/or confer a selective growth advantage promote successive waves of subclonal expansion depending on local conditions and/or exposure to therapeutic agents. Such a complex history of subclonal evolution leading to the production of billions of genetically heterogeneous cells in human breast cancers has been dramatically revealed from genomic DNA sequence data (Nik‐Zainal *et* *al,* 2012; Eirew *et* *al,* 2014). And this profound inter‐tumor as well as intra‐tumor heterogeneity is further exacerbated by the metastatic process in which subclones differentially populated different sites.

Breast cancers are currently classified clinically on the basis of their extent and confinement, or not, within the basement membrane that surrounds the normal mammary gland, the proliferative activity and presence of nuclear abnormalities in the malignant cells, and their expression of ER, PR, and HER2. Global gene expression profiling has led to the identification of five major subtypes (Perou *et* *al,* 2000) that can now be distinguished based on the measurement of transcript levels of just 50 genes (PAM50; Parker *et* *al,* 2009; Nielsen *et* *al,* 2010; Chia *et* *al,* 2012). Notably, many of these detect the same perturbed features that have long been recognized histologically (Table 1). The five major subtypes thus identified are referred to as follows: basal‐like, luminal A, and luminal B, normal‐like, and claudin‐low tumors. More recently, additional subdivisions have come from analyses of both genomic sequencing data (Cancer Genome Atlas Network, 2012; Curtis *et* *al,* 2012) and altered epigenomic marks (Holm *et* *al,* 2010, 2016; Kamalakaran *et* *al,* 2011).

**Table 1:** List of the genes used for the PAM50 classification

Symbol	Histology	BC vs. LP	BC vs. LC	LC vs. LP
ACTR3B				
ANLN				
BAG1				
BCL2			DN	UP
BIRC5				
BLVRA				UP
CCNB1				
CCNE1				
CDC20				
CDC6				
CDH3				
CENPF				
CEP55				
CXXC5				UP
EGFR			UP	DN
ERBB2	**✓**			
ESR1	**✓**	DN	DN	
EXO1				
FGFR4				
FOXA1			DN	UP
FOXC1				DN
GPR160			DN	UP
GRB7				
KIF2C				
KRT14		UP	UP	
KRT17		UP	UP	
KRT5		UP	UP	DN
MAPT				
MDM2				
MELK				
MIA		DN		DN
MKI67	**✓**			
MLPH		DN	DN	UP
MMP11				
MYBL2				
MYC				
NAT1			DN	UP
NDC80				
NUF2				
ORC6				
PGR	✓			UP
PHGDH				DN
PTTG1			UP	
RRM2				
SFRP1			UP	DN
SLC39A6				UP
TMEM45B			DN	UP
TYMS				
UBE2C				
UBE2T				

Gene products used routinely in histological studies (✓) and transcripts increased (UP) or decreased (DN) in mammary epithelial cells are marked. Differential gene expression data are based on the RNA‐seq data presented in Pellacani *et* *al* (2016).

Interestingly, the expression profiles of the five main cancer subtypes are correlated with expression profiles of BCs, LPs, and LCs (Table 1). Even the PAM50 signature relies on an assessment of many gene transcripts (e.g., *FOXA1, PGR, ESR1, KRT14, KRT5, EGFR, FOXC1*, and *MIA*) that are normally present at different levels in BCs, LPs, and LCs. Generally, the transcriptional profiles of basal‐like breast cancers are closest to those of LPs, those of luminal A and B cancers to LCs, and claudin‐low cancers to BCs. These findings reinforce the concept that malignancies represent perturbations of the normal tissue from which they arise and frequently retain many components of the transcriptional regulatory networks that control cell production, differentiation, and death in the normal human mammary gland.

## Altered transcriptional regulation in human breast cancers

Breast cancer “drivers” is a term that has been used to refer to mutations that are found repeatedly, suggesting they contribute to the malignant properties of the cells. In contrast, “passenger mutations” is a term often assigned to mutations that are rare and do not appear to be relevant to the genesis or progression of the malignant population. It is notable that a majority of the most frequently encountered mutations affect genes linked directly or indirectly to transcriptional regulation (Nik‐Zainal *et* *al,* 2016; Zacksenhaus *et* *al,* 2017).

**Figure 5:**
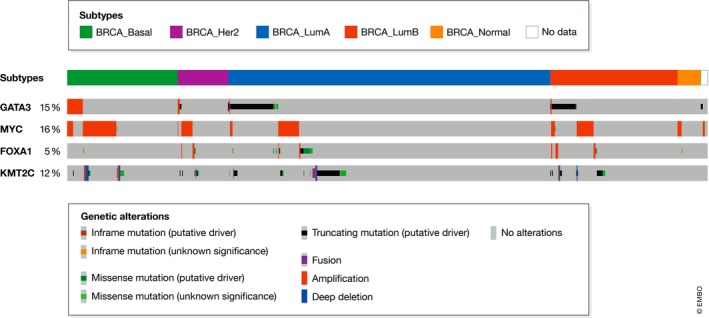
Frequency of genomic alteration of *GATA3*,* MYC*,* FOXA1,* and *KMT2C* in human breast cancer subtypes Heatmap showing the frequency of genomic alterations detected in *GATA3*,* MYC*,* FOXA1,* and *KMT2C* in human breast cancer subtypes. Data are drawn from the 993 breast cancer cases in the TCGA PanCancer Atlas study analyzed and plotted via cBioportal (http://www.cbioportal.org).

One of the most frequently altered transcriptional regulators is *GATA3* (mutated in > 10% of cases; Cancer Genome Atlas Network, 2012), most often in ER^+^ breast cancers (Fig 5; Nik‐Zainal *et* *al,* 2016). Both clinical and experimental lines of evidence link mutations in *GATA3* directly to breast cancer development and progression. Expression of GATA3 has been associated with a favorable prognosis, although this is still debated (Chou *et* *al,* 2010; Takaku *et* *al,* 2018), and similarly, in mice and cell lines, a heightened expression reduces tumorigenesis, suppresses metastasis, and promotes expression of a luminal molecular signature. In contrast, a loss of GATA3 has been found to accelerate tumor progression (Asselin‐Labat *et* *al,* 2011; Chou *et* *al,* 2013).

In > 15% of breast cancers, *MYC* is amplified. This is generally associated with an unfavorable clinical prognosis (Deming *et* *al,* 2000) and an ER^−^ breast cancer phenotype (Fig 5; Nik‐Zainal *et* *al,* 2016). MYC is one of the most intensively studied oncogenes (Fallah *et* *al,* 2017). Of particular note is recent evidence that overexpression of MYC in immortalized human mammary cells triggers a reprogramming of the epigenome that confers tumor‐initiating proprieties and a down‐regulation of luminal‐specific TFs and genes (Poli *et* *al,* 2018). MYC activity has also been shown recently to be influenced by its interaction with *EPIC1,* a long non‐coding RNA, that is upregulated in many cancers (Wang *et* *al,* 2018). Interestingly, MYC amplification was also reported to be a frequent event in the genesis of transformants from primary human mammary cells (Elenbaas *et* *al,* 2001) and in radiation‐induced mammary cell lines (Wade *et* *al,* 2015).

*FOXA1* is a TF that is mutated or amplified less frequently in human breast cancers (~ 2% mutated and ~ 1% amplified; Cancer Genome Atlas Network, 2012; Robinson *et* *al,* 2013) and usually found to be altered in ER^+^ tumors (Fig 5; Nik‐Zainal *et* *al,* 2016). FOXA1 is a main regulator of steroid receptor function in cancer (Augello *et* *al,* 2011), and it regulates ER signaling in breast cancer (Carroll *et* *al,* 2005; Lupien *et* *al,* 2008). FOXA1 mediates ESR1 binding and transcriptional activity (Hurtado *et* *al,* 2011), and its expression is associated with superior breast cancer outcomes (Shou *et* *al,* 2016). Molecularly, FOXA1 can recruit KMT2C (MLL3) to deposit H3K4me1 on FOXA1‐bound enhancers (Jozwik *et* *al,* 2016).

*KMT2C* is another frequently mutated transcriptional regulator in breast cancer, with a mutational spectrum consistent with a loss‐of‐function role (Wang *et* *al,* 2011; Ellis *et* *al,* 2012; Cancer Genome Atlas Research Network, 2015). Functionally, it is the catalytic component of a complex called COMPASS (complex of proteins associated with Set1) or ASCOM (ASC‐2‐ and MLL3‐containing complex) and responsible for the monomethylation of H3K4 (Herz *et* *al,* 2014). In mouse models, *Mll3* deletion in the mammary gland results in hyperplasia and expansion of cells with basal features in transplant experiments, and an acceleration of PI3K‐driven tumorigenesis (Zhang *et* *al,* 2016), supporting its role as a tumor suppressor.

Many other histone methyltransferases are deregulated in breast cancer by genetic alteration (Michalak & Visvader, 2016) and thereby contribute to an increased emergence of epigenomic alterations in breast cancer. Consequent changes in the epigenomes of analyzed human breast cancers have revealed more than 100 frequently hyper‐ or hypo‐methylated gene promoters and pronounced global DNA hypo‐methylation (Davalos *et* *al,* 2017; Pasculli *et* *al,* 2018), and the functional implication of these changes is now starting to be investigated using CRISPR/Cas9 systems (Saunderson *et* *al,* 2017). These findings are particularly interesting clinically, as they may offer new biomarkers of risk, prognosis, and treatment response (Pouliot *et* *al,* 2015; Terry *et* *al,* 2016) that can be robustly measured at relatively low cost (Cheuk *et* *al,* 2017). However, downstream transcriptional alterations are not consistently predicted and many exceptions to the general inverse correlation between promoter methylation and gene expression exist. In addition, expression of many frequently hypermethylated genes in breast cancer cells is already repressed in normal mammary cells, usually by polycomb group proteins depositing H3K27me3 (Sproul *et* *al,* 2011).

Comparisons of the DNA methylation profiles of individual breast cancers have shown they are highly heterogeneous. However, when subjected to unsupervised clustering, these profiles subdivide into groups that correspond largely to established transcriptionally defined breast cancer subtypes with corresponding similarities to normal human mammary subpopulations (Holm *et* *al,* 2010, 2016; Kamalakaran *et* *al,* 2011). From these, specific DNA methylation signatures with prognostic potential have been derived for luminal B and basal‐like subtypes (Stefansson *et* *al,* 2015).

Interestingly, DNA sequence alterations that do not occur within regions that encode protein sequences directly (non‐coding mutations) represent ~ 98% of mutations in cancer and most still remain poorly characterized. Of these, mutations occurring in cis‐regulatory elements (i.e., enhancers and promoters) are of particular interest, as they can directly alter expression of associated gene products, by directly or indirectly altering DNA binding of TFs (Deplancke *et* *al,* 2016; Shi *et* *al,* 2016). Such mutations are frequent in breast cancer (Bailey *et* *al,* 2016; Zhou *et* *al,* 2016; Rheinbay *et* *al,* 2017; Gyorffy *et* *al,* 2018), but their significance is generally unclear (Nik‐Zainal *et* *al,* 2016). However, mutations in *ESR1* enhancer sequences found in ~ 7% of breast cancers have now been shown to be responsible for altering *ESR1* expression by modulating TF binding activity (Bailey *et* *al,* 2016). In addition, a single‐nucleotide variant in one of these enhancer sequences has been associated with increased breast cancer risk. Mutations in the promoter of *FOXA1* that cause its overexpression through increased E2F binding constitute a second documented example of a biologically relevant mutation in a cis‐element in some breast cancer genomes (Rheinbay *et* *al,* 2017). Variants linked to increased breast cancer risk have been found in distal regulatory elements of genes whose expression is modulated by FOXA1 (Cowper‐Sal Lari *et* *al,* 2012).

Breast cancers also contain many cell types that are not part of the malignant population but, nevertheless, interact with them and co‐evolve with them, adding further to the complexity and heterogeneity of breast tumors (Hanahan & Weinberg, 2011). These additional cell types include components of the blood and lymph vasculature, tissue macrophages and lymphocytes, and various stromal fibroblasts and their derivatives. Both the infiltrating leukocytes and resident cancer‐associated fibroblasts (CAFs) are now well established as playing significant roles in modulating breast cancer cell growth and plasticity through direct interactions as well as through their secretion of growth factors, cytokines, and extracellular matrix components (Allinen *et* *al,* 2004; Aboussekhra, 2011; Place *et* *al,* 2011; Esquivel‐Velázquez *et* *al,* 2015; Qiao *et* *al,* 2016).

One of the best characterized mechanisms of CAF modulation of human breast cancer cells is mediated by their secretion of TGF‐β. Recently, this has been updated to include the suppression of adjacent normal mammary cells (Chatterjee *et* *al,* 2018) and the promotion of EMT in a xenografted breast cancer cell line through the transactivation of a HOX transcript antisense RNA (Ren *et* *al,* 2018). A third recently described role of CAFs is their induction of a FOXA1‐mediated creation of a hormone‐sensitive, luminal gene regulatory program in basal‐like breast cancers in response to PDGF secretion by the tumor cells (Roswall *et* *al,* 2018). Loss of TP53 in stromal fibroblasts has also been shown to promote breast tumor development *in vivo* through the production of SDF‐1 (Addadi *et* *al,* 2010). Additional reported mechanisms include the altered expression in CAFs of non‐coding RNAs and microRNAs (Verghese *et* *al,* 2013; Shah *et* *al,* 2015; Ren *et* *al,* 2018). Other components of the tumor microenvironment, including tumor‐associated macrophages, have been implicated in tumor promotion through the expression of *TFEB* (Fang *et* *al,* 2017).

## Transcriptional deregulation during the initiation of breast cancers

Early events important to the genesis of human breast cancer are still limited and largely extrapolated from transgenic mouse models. Information derived from studies of human cancers has been largely limited to retrospective analyses of prevalent changes in established tumors (Futreal *et* *al,* 2004; Nik‐Zainal *et* *al,* 2012), or a few analyses of preneoplastic mammary cells were obtained from carriers of *BRCA1* mutations (Lim *et* *al,* 2009; Proia *et* *al,* 2011; Choudhury *et* *al,* 2013) or from samples of ductal carcinoma *in situ* (DCIS; Yeong *et* *al,* 2017). Events that accompany the acquisition of malignant properties by immortalized, but non‐tumorigenic, human mammary cell lines have also been described (Debnath *et* *al,* 2003; Leung & Brugge, 2012). More recently, experimental models initiated directly with primary human mammary cells have been reported.

Transgenically controlled overexpression of potential culprit genes in mice, including overexpression of MYC and HER2, was important in providing the first experimental evidence that oncogene overexpression alone could induce the formation of malignant tumors (Stewart *et* *al,* 1984; Muller *et* *al,* 1988; Bouchard *et* *al,* 1989). Since then, derivative approaches are now able to model metastasis due to expression of co‐operating oncogenes (Sinn *et* *al,* 1987; Guy *et* *al,* 1992; Podsypanina *et* *al,* 2008; Adams *et* *al,* 2011) and assess mechanisms of pathway perturbation including TGF‐β and WNT (Pierce *et* *al,* 1995; Li *et* *al,* 2000). The introduction of conditional and inducible systems to drive the expression of transgenes has enabled these models to be further refined (Sandgren *et* *al,* 1995; Moody *et* *al,* 2002; Podsypanina *et* *al,* 2008; Menezes *et* *al,* 2014; Rutkowski *et* *al,* 2014), including a model in mice of invasive lobular breast cancer created using CRISPR/Cas9‐mediated disruption of PTEN (Annunziato *et* *al,* 2016).

However, a major criticism of these mouse models of breast cancer is the very ease with which the tumors can be generated. They also frequently lack the genetic complexity of human breast cancers, and their similarities to their human counterparts are often restricted to specific sites within the tumors produced (Cardiff *et* *al,* 2000; Hollern *et* *al,* 2018). In addition, their pathology may be highly dependent on the promoters used to drive expression of the oncogenic transgene and few display highly invasive properties (Cardiff *et* *al,* 2000). Gene expression differences in mice are also notable (Pfefferle *et* *al,* 2013), and some types of human breast cancer have not yet been possible to model in mice. For example, although ER^+^ tumors account for the majority of all human breast cancers, stably ER^+^ mouse mammary tumors have been difficult to obtain and the genetic changes that lead to ER expression in mouse tumors are frequently not characteristic of patients’ ER^+^ tumors (Mohibi *et* *al,* 2011).

Immortalized cell lines, and the MCF10A line in particular, have also been used for modeling the human mammary cell transformation process also because of their ease of use and manipulation and their availability in virtually unlimited numbers. MCF10A cells were generated by immortalizing human mammary cells obtained from a donor with benign fibrocystic disease (Soule *et* *al,* 1990). Forced expression of multiple cancer genes in these cells has been found to induce some features of transformation (recently reviewed in Balani *et* *al,* 2017). Notably, aggressively tumorigenic lines have been derived from MCF10A cells forced to overexpress HRAS and passaged *in vivo,* and their extensive characterization has revealed the presence of a number of predicted driver mutations (Maguire *et* *al,* 2016). However, their controlled modification has not recapitulated the phenotypic, genomic, and functional heterogeneity found in most spontaneously arising human breast cancers (Kaur & Dufour, 2012).

Analysis of DCIS has been another strategy used to investigate early events leading to invasive breast cancer. Initial transgenic mouse models of DCIS were obtained by driving expression of the SV40 large tumor antigen with the mouse WAP promoter that becomes highly active in terminal lobular luminal cells in pregnant mice (Schulze‐Garg *et* *al,* 2000). More recently, *in vivo* models of human DCIS have also been developed by the intraductal injection of mice with experimentally transformed human cell lines (Behbod *et* *al,* 2009) or primary DCIS samples from patients (Valdez *et* *al,* 2011). These models generally recapitulate the histology and heterogeneity of the human disease, including occasional examples of disease progression indicated by cellular invasion into the surrounding stroma.

Experimental models of *de novo* mammary tumorigenesis starting from isolated primary cells from normal tissues are particularly attractive because they avoid species differences and concerns of extrapolating from human immortalized cell lines. However, there are very few reports of genetic perturbations that consistently yield fully malignant human mammary cells in transplanted female immunodeficient mouse hosts (either NOD/SCID or NRG—NOD‐*Rag1* ^−/−^‐*IL2Rγc* ^−/−^ mice). Interestingly, most of those that have been reported have used different combinations of oncogenes, cell types, and sites of injection, with or without added fibroblasts. Immunohistological analyses of tumors produced from human EpCAM^+^ luminal cells transduced with either TP53^R175H^ + CCND1 + myristoylated PIK3CA + KRAS^G12V^ or SV40 T antigen + KRAS^G12V^ transplanted into “humanized” fat pads of NOD/SCID mice (obtained by added injection of human fibroblasts) suggested the tumors most closely resemble ductal carcinomas with predominant luminal features, including expression of ERα, CK8/18, and CK19. In contrast, the same manipulation of CD10^+^ (basal) cells caused them to acquire squamous and metaplastic features with reduced ERα and CK19 expression and robust expression of the basal marker, CK14 (Keller *et* *al,* 2012). On the other hand, we have found that transduction of either normal human BCs or LPs (but not LCs or SCs from the same mammoplasty samples) with just a *KRAS* ^*G12D*^‐encoding vector produces serially transplantable invasive ductal carcinomas rapidly and at high efficiency in mice using injection sites under the kidney capsule or subcutaneously (Nguyen *et* *al,* 2015). These *KRAS* ^*G12D*^‐derived tumors are also highly heterogeneous with variable proportions of cells positive for ERα, Ki67, EGFR, CK14, and CK8/18, independently of their BC or LP cell of origin (Nguyen *et* *al,* 2015).

Use of a DNA barcoding strategy, to track the clonal dynamics of the primary and secondary *KRAS* ^*G12D*^‐derived tumors, showed them to be consistently and highly polyclonal, regardless of the initial cell type transduced (Nguyen *et* *al,* 2015). The median size of the few clones found in both primary and secondary tumors derived from the same initial inoculum was larger than most of the clones appearing only after a first passage. Interestingly, normal human mammary cells transduced with the same tracking vector also showed a delayed appearance of new and larger clones in the “normal” structures obtained in secondary as compared to primary recipients of the same original cells (Nguyen *et* *al,* 2014). The invasive nature of the primary clones but their general lack of perpetuation in secondary implants contrasts with the conventional concept of the oncogenic process, in which the control of invasive properties by human mammary cells is usually modeled as property that is acquired *after* deregulated growth has created a large “premalignant” population from which a more advanced derivative then arises. Taken together, these findings thus challenge previous assumptions of a requirement for a multi‐step selective process during which the genetic and/or epigenetic changes needed to obtain a continuously growing invasive tumor are successively accrued.

Transcriptional profiling of the polyclonal *KRAS* ^*G12D*^‐induced primary tumors we have described has shown they are characterized by a global deregulation of gene expression that is largely but not completely independent of the cell type used to initiate them (Nguyen *et* *al,* 2015). A similar result was found for tumors derived by transducing primary cells from the same normal donors with SV40 T antigen + KRAS^G12V^ (Keller *et* *al,* 2012) or cells from donors with a different *BRCA1* mutation status using vectors encoding TP53^R175H^ + CCND1 + myristoylated PIK3CA + KRAS^G12V^ (Proia *et* *al,* 2011). Thus, the initiating cell type may not necessarily make a major contribution to the transcriptional profile of the cells constituting the bulk of any breast cancer. Such a concept is of interest as it challenges the idea that globally acquired molecular profiles of breast cancers will provide informative indications of the cell of origin or the cells from which relapses are most likely to emerge.

## Conclusions

Heterogeneity is a pronounced feature of human breast cancer genomes and epigenomes. These variable features likely explain the corresponding heterogeneity evident in the transcriptomes of these malignant populations. The multitude of these alterations, plus the still partial elucidation of the molecular networks governing the properties of normal human mammary cells, still obscures identification of critical initial transforming events. Nevertheless, early changes that lead to human breast cancer development remain important potential targets for more effective strategies. Expansion of *de novo* models now appears possible with established robust transduction protocols and new screening approaches on the horizon. The coupling of these strategies with clonal analyses, highly multiplexed gene manipulations, and exposure to small molecules thus holds new promise for the future more rapid identification of targetable mechanisms critical to breast cancer development.

## Conflict of interest

The authors declare that they have no conflict of interest.
